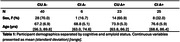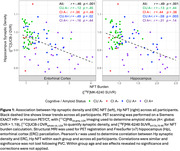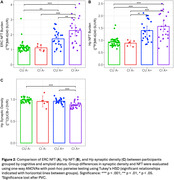# Association of synaptic density and neurofibrillary tau in preclinical and clinical Alzheimer’s disease

**DOI:** 10.1002/alz.090399

**Published:** 2025-01-09

**Authors:** Alexandra H DiFilippo, Bradley T. Christian, Erin M. Jonaitis, Max McLachlan, Andrew K McVea, Brecca Bettcher, Nicholas R Schulz, Mary‐Elizabeth Pasquesi, Nancy J Davenport‐Sis, Yer Thor, Ethan Grover, Todd E Barnhart, Jonathan W Engle, Tobey J. Betthauser, Sterling C. Johnson, Barbara B. Bendlin

**Affiliations:** ^1^ University of Wisconsin School of Medicine and Public Health, Madison, WI USA; ^2^ Waisman Center, University of Wisconsin‐Madison, Madison, WI USA

## Abstract

**Background:**

Synaptic density loss is a major correlate of cognitive functioning in adults with and without significant impairment. It is also a feature of clinical Alzheimer’s disease (AD) and suspected to co‐occur with neurofibrillary tau (NFT) accumulation. Previous studies using [^11^C]UCB‐J for in‐vivo analysis of synaptic density have been restricted to unimpaired (CU) A‐ and impaired (CI) AD (A+) participants where within group analyses have shown no association between synaptic density and NFT. There is also little known about the relationship between synaptic density and NFT in AD without cognitive impairment. Here we evaluated the association between synaptic density and NFT in CU and CI AD.

**Method:**

Participants were recruited from ongoing AD‐related studies (Wisconsin Registry for Alzheimer’s Prevention, ADRC) at UW and from the community and underwent comprehensive clinical and cognitive evaluation to determine cognitive status. Pearson’s *r* was used to determine associations between synaptic density ([^11^C]UCB‐J DVR) and neurofibrillary tau ([^18^F]MK‐6240 SUVR) across all participants and within amyloid and cognitive groups.

**Result:**

Participant demographics are reported in Table 1. Across all participants, synaptic density and ERC, Hp NFT were well correlated (ERC NFT: *r* = ‐.46, *p* < .001; Hp NFT: *r* = ‐.49, *p* < .001). Within groups, only the CU A+ correlation between synaptic density and NFT remained significant (ERC NFT: *r* = ‐.49, *p* = .02; Hp NFT: *r* = ‐.59, *p* = .003) with CI A+ showing only a moderate (insignificant) relationship with Hp NFT (ERC NFT: *r* = ‐.16, *p* = .44; Hp NFT: *r* = ‐.31, *p* = .14) (Figure 1).

**Conclusion:**

In agreement with previous work, CI AD has lower Hp synaptic density than CU A‐, and no significant relationship between Hp synaptic density and NFT. However, CU AD participants showed a significant relationship between NFT and synaptic density. This may be explained by a floor effect of Hp synaptic density that is reached by participants with clinical AD, but not yet in preclinical AD, or the existence of non‐AD pathologies contributing to synapse loss. These results can help better understand the process of neurodegeneration in AD as participants progress from preclinical to clinical AD.